# Structural changes of mesophyll cells in the rice leaf tissue in response to salinity stress based on the three-dimensional analysis

**DOI:** 10.1093/aobpla/plae016

**Published:** 2024-04-23

**Authors:** Rachana Ouk, Takao Oi, Daisuke Sugiura, Mitsutaka Taniguchi

**Affiliations:** Graduate School of Bioagricultural Sciences, Nagoya University, Nagoya 464-8601, Japan; Graduate School of Bioagricultural Sciences, Nagoya University, Nagoya 464-8601, Japan; Graduate School of Bioagricultural Sciences, Nagoya University, Nagoya 464-8601, Japan; Graduate School of Bioagricultural Sciences, Nagoya University, Nagoya 464-8601, Japan

**Keywords:** 3D, chloroplast, intercellular airspace, leaf tissue, mesophyll cell, salt stress, serial section light microscopy

## Abstract

Rice leaf blades have intricate-shaped mesophyll cells (MCs) with a large volume of chloroplasts enhancing gas exchange between stroma and intercellular airspace (IAS). Since the rice MCs do not form palisade or spongy tissue cells and are considered monotypic cells, the structural analysis of MCs in the middle part of the leaf tissue has been done, neglecting the various shapes of MCs can be observed on the cross-section of rice leaves. Moreover, the middle MC layer is sandwiched between the upper and lower layers and is more restricted in its demand for light and CO_2_ entering from the outside. Therefore, the different layers of MCs may differ in their sensitivity to salt stress that causes structural changes in cells. This study aims to elucidate the intra- and extra-cellular structures of MC in different layers of leaf tissue and determine how salinity affects the MC structure in each layer. The mesophyll tissue was divided into adaxial, middle and abaxial layers, and eight MCs and chloroplast regions were selected from each layer and reconstructed into three-dimensional (3D) representations. The whole leaf anatomical and physiological parameters were measured to find the effects of salinity stress on the MC structures. As a result, the 3D analysis of rice leaf tissue revealed the different structures of MCs with greater diversity in the adaxial and abaxial layers than in the middle layer. Salinity stress reduced the size and height of the MCs and coverage of the chloroplast on the cytoplasm periphery of the adaxial and abaxial layers, as well as the chloroplast size of adaxial MCs. Overall, these results reveal the variation of rice MC in leaf tissue and suggest the higher sensitivity to salt stress in the adaxial mesophyll among the layers, which may partly account for the decrease in photosynthetic capacity.

## Teaser

This study focuses on the diverse structures of rice leaf mesophyll cells (MCs) across different layers and investigates their response to salinity stress. While middle-layer MCs are traditionally studied, our three-dimensional analysis reveals greater diversity in adaxial and abaxial layers. Salinity stress disproportionately affects adaxial MCs, reducing size and chloroplast coverage. These findings emphasize layer-specific variations in structural sensitivity, providing insights into the nuanced responses of rice MCs to environmental stressors.

## Introduction

Leaves are the main sites of photosynthetic activity, influencing plant growth and productivity. Photosynthesis is primarily related to the mesophyll layers with the number of chloroplasts (Lehmeier *et al.* 2017). Furthermore, the mesophyll tissue structure influences the photosynthetic activity (Flexas *et al.* 2008) and affects the leaf hydraulic traits (Sack *et al.* 2003). The mesophyll architecture varies according to species and growth environments, such as temperature, light, water and nutrition, which alter the mesophyll structure and cause a change in photosynthetic activity. Therefore, understanding the mesophyll structure is one of the essential ways to enhance plant productivity and photosynthetic features (Giuliani *et al.* 2013).

Leaf anatomy has been studied frequently using two-dimensional (2D) microscopic images to understand the physiological roles of leaf tissues. In the last decades, the three-dimensional (3D) reconstruction technique has been used in plant science and revealed the leaf anatomical traits (Earles *et al.* 2018). The serial cross-sectioning approach is one of the methods that are accessible to virtualize 2D section images into 3D reconstruction. Serial section electron microscopy (ssEM) has been used since the 1950s (Birch-Andersen 1955; Sjöstrand 1958) to the present (Yamane *et al.* 2018, 2022). With the advantage of light microscopy (LM) to distinguish intracellular organelles and substances by staining sections, the semithin serial section LM (ssLM) using a diamond knife with a large boat assists in getting the perfect alignment of the section with the identical orientation of the structure and the completeness of the series, which is advantageous for the 3D reconstruction (Blumer *et al.* 2002). Recently, we established the 3D reconstruction method based on double-stained ssLM, which can detect not only mesophyll cell (MC) and chloroplasts at the cellular level but also at the leaf tissue level, in addition to calculating anatomical traits (Ouk *et al.* 2020, 2022).

The structure of each MC defines the patterning of intercellular airspace (IAS) in a leaf, which is considered to have high CO_2_ diffusion conductance (Lehmeier *et al.*, 2017; Earles *et al.*, 2018). The armed MCs of grass species with lobes have been suggested to improve the MC surface area facing IAS (*S*_mes_), increasing the pathway for CO_2_ diffusion to chloroplasts (Terashima *et al.* 2011). Furthermore, the arrangement of chloroplasts adjusted to the MC wall increases the chloroplast surface area facing IAS (*S*_c_), explaining the high CO_2_ transfer conductance to the photosynthetically active sites (Caemmerer and Evans 1991; Parkhurst, 1994). Rice is well known for having armed MCs with a large volume of chloroplasts enhancing the gas exchange between stroma and IAS (Sage and Sage, 2009; Adachi *et al.*, 2013; Oi *et al.*, 2017). Although grasses show no distinct palisade and spongy parenchyma, the profile of rice MCs differs depending on their location in the leaf tissue (Hoshikawa 1989). It is reported that there are two types of MCs in the rice leaf tissue; MCs with wide and elongated arms on the cell periphery and those with short arms and fewer projections (Chonan 1970). However, the former type appeared more frequently in the transverse sections. Therefore, the rice MCs are assumed as monotypic cells, and there are few studies focussing on variation in MC structure within the whole leaf tissue.

Furthermore, the leaf structure and chloroplast ultrastructure are affected by abiotic stresses such as heat, drought and salinity. Salinity is one of the major environmental stresses limiting plant photosynthesis, growth and productivity (Boyer 1982; Flexas *et al.* 2004). The combinations of osmotic and ionic phases in salinity stress result in rapid inhibition of plant growth and high Na^+^ and Cl^−^ accumulations in leaf tissue (Munns and Tester 2008). Moreover, these combinations reduce the activity of many enzymes (Allakhverdiev *et al.* 2000) and induce cellular dehydration (Boughalleb *et al.* 2009), the latter of which could cause the cell wall thickening and the decreases in mesophyll conductance and CO_2_ assimilation (Flexas *et al.* 2021). Especially in the MCs, the most sensitive organelle to salinity stress are chloroplasts (Rahman *et al.*, 2000; Mitsuya *et al.*, 2003; Yamane *et al.*, 2008). Recently, the electron and light microscopies followed by 3D reconstruction revealed the intricated shape of the whole MC in rice leaves and detected the deformation of the chloroplasts in response to salinity stress (Oi *et al.* 2020; Ouk *et al.* 2020) although both studies focussed only on the middle part of the rice leaf tissue. However, MCs close to the adaxial and abaxial epidermis receive different light intensities (Terashima *et al.* 1986, 2009; Takahashi *et al.* 1994) and show asymmetrical photosynthetic characteristics (Kumagai *et al.* 2014). Therefore, localization of the two types of MCs in rice leaf tissue (Chonan 1970) and changes in MC structures in response to salinity stress along the adaxial–abaxial axis should not be neglected. Here, we hypothesized that the rice MCs and chloroplasts in the adaxial layer have a more suitable structure for photosynthesis but are more susceptible to salinity stress. Since the adaxial surface directly faces the light, it is favourable for photosynthesis under non-stressed conditions; however, light is a major factor in oxidative stress causing changes in chloroplast thylakoid structure (Scandalios 1993; Bondada & Oosterhuis 1998). The combination of salinity stress and excess light could cause more damage to mesophyll cells and their chloroplasts at the adaxial side, resulting in a decrease of *S*_mes_ and *S*_*c*_ that reduces CO_2_ absorption and photosynthetic activity.

In this study, we aim to elucidate the variation of MC structures and determine how salinity affects the structure of rice leaf tissue using the ssLM followed by the 3D reconstruction method (Ouk *et al.* 2022), which is able to analyse the 3D anatomy of the whole leaf tissue. The mesophyll tissues were divided into adaxial (under the upper epidermis), abaxial (above the lower epidermis) and middle layers (between the two layers). The morphological and physiological parameters were measured and discussed to find the effects of the salinity stress on the structure of MCs.

## Materials and Methods

### Plant materials and growth conditions

Caryopsis of rice (*Oryza sativa* ‘Nipponbare’) were soaked in distilled water and incubated in a growth chamber at 28 °C/20 °C (day/night) until the white tip of the coleoptile appeared, the seedlings were transplanted onto a mesh above a plastic bucket containing tap water and grown in a growth chamber under a 14 h photoperiod (8:00 to 22:00 h) at 400–500 µmol m^–2^ s^–1^ and 28/20 °C (day/night). Two days later, the tap water was changed to the nutrient solution of Mae & Ohira (1981) contained (mM): NH_4_NO_3_, 1.0; NaH_2_PO_4_-2H_2_O, 0.6; K_2_SO_4_, 0.3; CaCl_2_-2H_2_O, 0.3; MgCl_2_-6H_2_O, 0.6; EDTA-Fe, 4.5 × 10^−2^; H_3_BO_3_, 5 × 10^−2^; MnSO_4_-5H_2_O, 9 × 10^−3^; CuSO_4_-5H_2_O, 3 × 10^−4^; ZnSO_4_-7H_2_O, 7 × 10^−4^; Na_2_MoO_4_-2H_2_O, 1 × 10^−4^ and all solutions were changed once a week. At 21 days after transplanted, the plants had already fully expanded fifth leaves. Subsequently, the plants were treated with 100 mM NaCl in the nutrition solution as salt-treated or without NaCl as a control for 4 days.

### Plant growth measurement

The plants were harvested on the fourth day of the salt treatment (25 days old); hence the plants had reached the mature seedling stage with the sixth leaves fully expanded. The plants were divided into shoots and roots, then measured for shoot fresh weights, and then dried at 70 °C for 72 h. The dry weights were measured, and the shoot water content was calculated by subtracting the dry weights from the fresh weights.

### Gas exchange and chlorophyll fluorescence measurements

The middle parts of the fifth leaf blades of each plant, which were already fully expanded at the beginning of salinity stress exposure, were used for the measurements. Gas exchange measurements were conducted between 9:00 and 11:00 h with a portable photosynthesis measurement system (LI-6800, LI-COR, USA) on the leaves. The net photosynthetic rate (*A*) was measured at PPFD of 500 µmol m^–2^ s^–1^ and ambient CO_2_ concentration of 400 µmol mol^–1^ air, and leaf temperature of 25 °C. Stomatal conductance (*g*_*s*_) and intercellular CO_2_ concentration (*C*_*i*_) were also recorded.

After the photosynthesis measurements, the relative chlorophyll content of a leaf was estimated with a chlorophyll metre (SPAD-502; Konica-Minolta, Japan). The chlorophyll fluorescence was measured between 14:00 and 15:30 h at 25 °C with a portable chlorophyll fluorometer (PAM-2100, Walz, Germany) on the adaxial leaf surface. The middle part of the leaf blades was adapted in the dark for 30 min. Then the minimal and maximal fluorescence yield (*F*_0_, *F*_*m*_) was determined. The maximal quantum yield of PSII (*F*_*v*_/*F*_*m*_) was calculated as (*F*_*m*_‒*F*_*0*_)/*F*_*m*_.

### Serial section light microscopy (ssLM) and dividing layers of rice leaf tissue

The middle parts of the leaf blades of the control and salt-treated plants used for the photosynthesis measurements were chemically fixed and embedded in resin. Then serial longitudinal sections parallel to the leaf veins were prepared and observed, according to Ouk *et al.* (2020). The sampling from the plant body under light was done between 09:30 and 11:30 h. The serial sections (0.5 µm thick) placed on a glass slide were double-stained with thionine and acridine orange for the chloroplasts and cell walls, respectively. The images were captured using a light microscope (BX 51, Olympus, Japan) with a CMOS camera (DP21 or DP74, Olympus) as following condition; image size: 1600 × 1200 pixels (0.15 µm pixel^–1^) and 2448 × 1920 pixels (0.08 µm pixel^–1^); colour depth: 24-bit; image file format: TIFF. The serial image brightness and contrast were adjusted and aligned using Fiji software (Schneider *et al.* 2012). The stacked serial images of rice mesophyll tissues were divided into three equal layers. The upper/lower one-third of mesophyll tissues laid under/above both epidermises are defined as the ‘adaxial layer’ and ‘abaxial layer’, respectively. The ‘middle layer’ is the layer between the adaxial and abaxial layers. The processed image stacks were shown as orthogonal slide images based on volume rendering using Image-Pro 3D software (version 10, Media Cybernetics, USA).

### 3D reconstruction

To detect the effects of salinity on the leaf anatomy, the MCs and chloroplast regions were reconstructed at the tissue and cellular levels.

For the tissue level, the MC and the chloroplast regions of each layer, the IAS and chloroplast regions close to the cell membrane, which here considered as in contact with the cell wall, were extracted and reconstructed into 3D representations as described in Ouk *et al.* (2022) using Image-Pro 3D software. The IAS was extracted from the binarized IAS and MC; then, the pure MC was binarized for the chloroplast regions. To obtain the chloroplast regions in contact with the cell wall, the chloroplast regions were dilated using the function ‘3D filters’ (width × height × depth = 7 × 7 × 1) and merged with the cell wall lines, which were extracted from the merged dilated MC (7 × 7 × 1) and the IAS.

At the cellular level, the MC, chloroplast regions and the cytoplasm periphery coverage were produced as described in Ouk *et al.* (2020). The eight MCs were randomly selected from each of adaxial, middle and abaxial layers. The cell walls of the MC were manually traced and painted using ‘PaintTool SAI’ software (version 1, Systemax, Japan) and were reconstructed into 3D representations using Image-Pro 3D software. The chloroplast regions of each MC were extracted from the traced MC and were dilated (7 × 7 × 1) to merge with traced MC cell wall lines to obtain the cytoplasm periphery coverage. The chloroplast regions and the cytoplasm periphery coverage were reconstructed into the 3D representations using the Image-Pro 3D software; the surface rendering representation was reconstructed with subsampling (512M voxel) and smoothing (low-pass filter [3:3:3]). The shape parameters (cell width, height, and depth, which is the length on the *x*, *y* and *z* axes, respectively) and the size parameters (volume and surface area) were calculated using the ‘3D Measure’ function.

### 3D calculation of leaf anatomical parameter

The IAS and the chloroplast regions in contact with cell walls obtained from the tissue level were used to calculate the 3D of *S*_mes_ and *S*_c_ as described in Ouk *et al.* (2022) in the following:

The surface area of MC facing IAS (*S*_mes_) in volume rendering sequence was calculated as:


$$S_{\text{mes}}=\frac{S_{\text{IAS}}}{\text{Leaf ​​}\;\text{​​ area}}\;(\mu m^{2}\mu m^{-2}),$$ (1)

where *S*_IAS_ is the surface area of IAS (µm^2^). Leaf area (µm^2^) is the product of the width of sections (µm) and the thickness of the stacked images (µm).

The surface area of chloroplast regions facing IAS (*S*_c_) in volume rendering sequence was calculated as:


$$S_{\text{c}}=\frac{S_{\text{chl}}}{\text{leaf ​​}\;\text{​​ area}}(\mu m^{2}\mu m^{-2}),$$ (2)

where *S*_chl_ is the surface area of chloroplast regions in contact with cell wall lines facing IAS (µm^2^).

Porosity is the measurement of the void space in the leaf tissue as the fraction of IAS volume per sum volumes of MC and IAS, as shown in the following equation:


$$\text{Porosity}=\frac{V_{\text{IAS}}}{V_{\text{MC ​​}\;\text{​​ }}+\;V_{\text{IAS}}}(\mu m^{3}\mu m^{-3}),$$ (3)

where V_IAS_ and V_MC_ are the total volumes of IAS and MC in leaf tissue, respectively.

### SEM observation

The serial section on the slide glasses was stained with 2 % uranyl acetate for 30 min, followed by lead citrate for 5 min, and coated with gold for 7 s using an ion-sputtering apparatus (IB-3; Eiko, Japan). The serial section was observed with a scanning electron microscope (SEM) (TM4000 PlusII, Hitachi, Japan) at an accelerating voltage of 5 kV. The wall thickness of MC was measured from electron micrographs. Sections of the cell wall were randomly selected, and thickness was measured using the Image-Pro 3D software.

### Statistical analysis

For the plant growth and physiological analysis, four plants were replicated for control and salt treatment. For the tissue analysis, three plants were used, and one leaf segment was taken per plant. For the cellular analysis, eight MCs from each layer were used. For cell wall thickness, three MCs from each layer were used. The data were statistically analysed using Microsoft Excel for Windows with the add-in software (Statcel 3, OMS Publishing Inc., Japan). The graphs show mean ± standard deviation (s.d.) with the result of the Student’s *t*-test or Tukey–Kramer multiple compared test followed by a two-way analysis of variance (ANOVA).

## Results

### The plant growth and photosynthetic parameters under salinity stress

**Figure 1.**
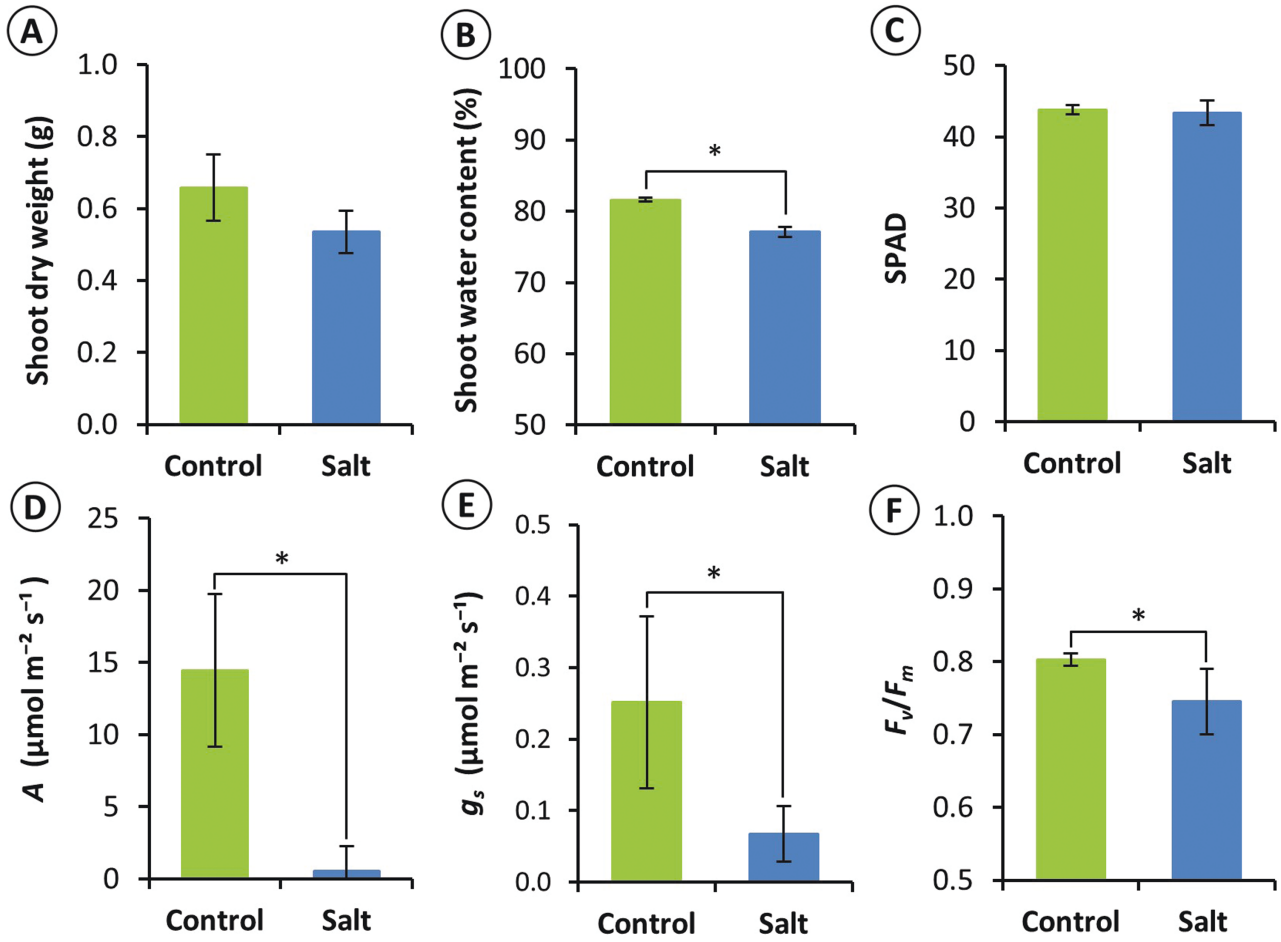
Growth and photosynthetic parameters after salt-treated. (a) Dry weight, (b) water content of shoot. (c) SPAD as chlorophyll content, (d) net photosynthetic rate (*A*), (e) stomatal conductance (*g*_*s*_), (f) *F*_*v*_/*F*_*m*_; maximal quantum yield of PSII of the fifth leaves. Mean ± s.d. (*n* = 4). Mean values between the control and salt-treated plants were compared by Student’s *t*-test (*P ˂ 0.05).

Four days after the treatment, the rice leaves in the control plants did not shrink and appeared green (Supporting Information—Fig. S1A), while the leaves in salt-treated plants shrunk, and one-third of the leaf tips turned yellow-brown (Supporting Information—Fig. S1B). Although the shoot dry weight showed no significant difference between the control and salt-treated plants (Fig. 1A), the shoot water content was significantly decreased in the salt-treated plants (Fig. 1B). The middle parts of the leaf blades were still green, and the SPAD values of the fifth leaves were not significantly different between the control and salt-treated leaves (Fig. 1C). However, *A* and *g*_*s*_ of the salt-treated plants were significantly decreased (Fig. 1D and E). The maximal quantum yield of PSII (*F*_*v*_/*F*_*m*_) was stable at around 0.8 in the control plants and significantly decreased to 0.75 in the salt-treated plants (Fig. 1F).

### Leaf anatomical properties in the layers of mesophyll tissues

**Figure 2.**
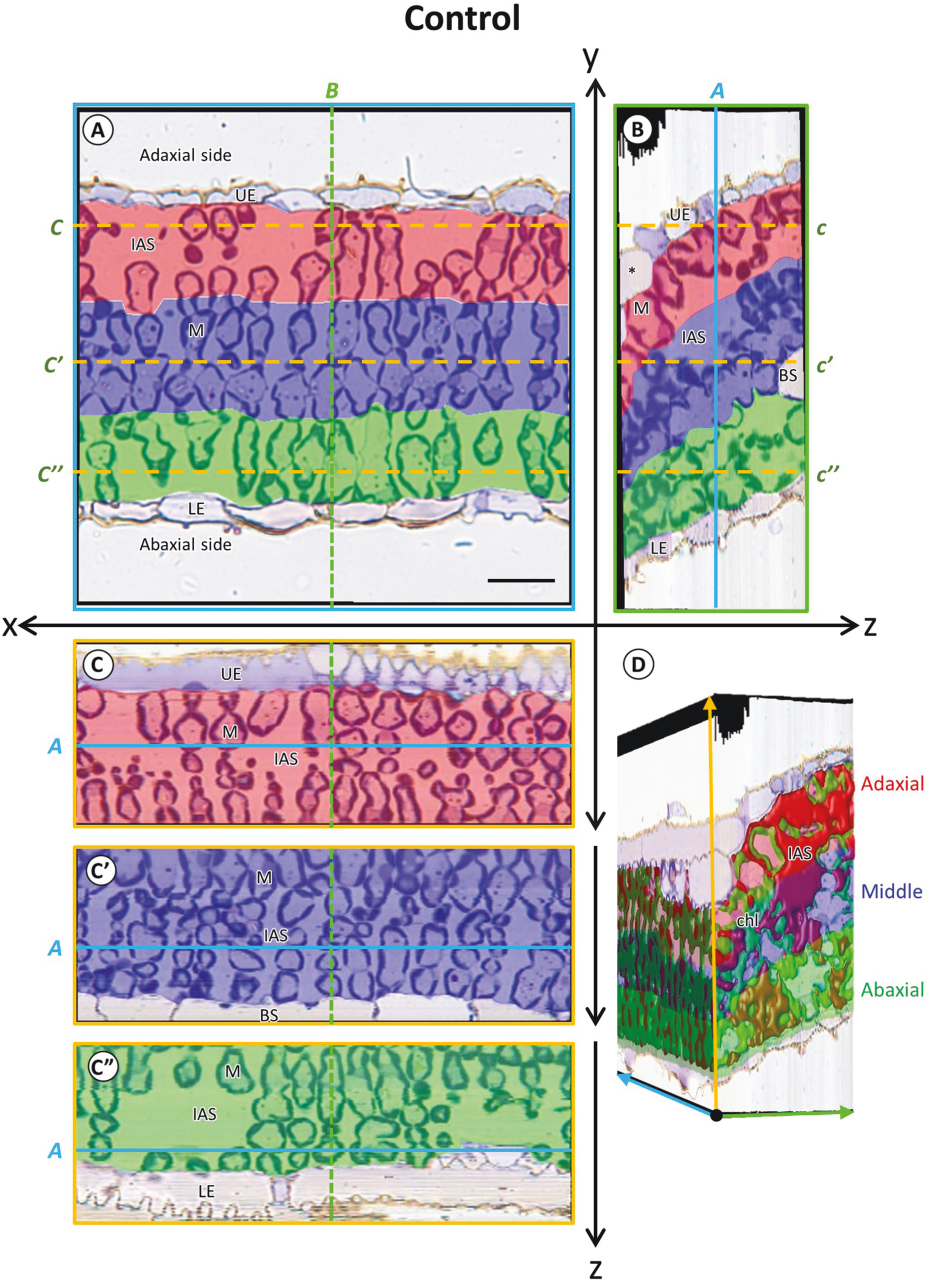
Orthogonal images of divided layers of mesophyll tissue of leaf blade in control rice. (A) Longitudinal image (*xy*). (B, C, Cʹ, C″) Orthogonal slice images of virtually imaged by volume rendering. (b) Transverse view (*yz*) of the image stacks, (C, Cʹ, C″) paradermal view (*xz*) of the image stacks. (D) Stacked view of the intercellular airspace and chloroplast in layers of leaf tissue. Each slice plane (A, B and C) crossed the lines marked as ‘*a, b, c*’ in other orientation images. Asterisk (*), motor cell, BS, bundle sheath cell; chl, chloroplast; IAS, intercellular airspace; LE, lower epidermis; M, mesophyll cells; UE, upper epidermis. The colour ‘Red, Blue, and Green’ in A–C″ indicated adaxial, middle and abaxial layers of MCs. Cutting interval (*z*-steps) = 0.5 µm. Total number of cuttings = 110. Scale bar = 20 µm.

**Figure 3.**
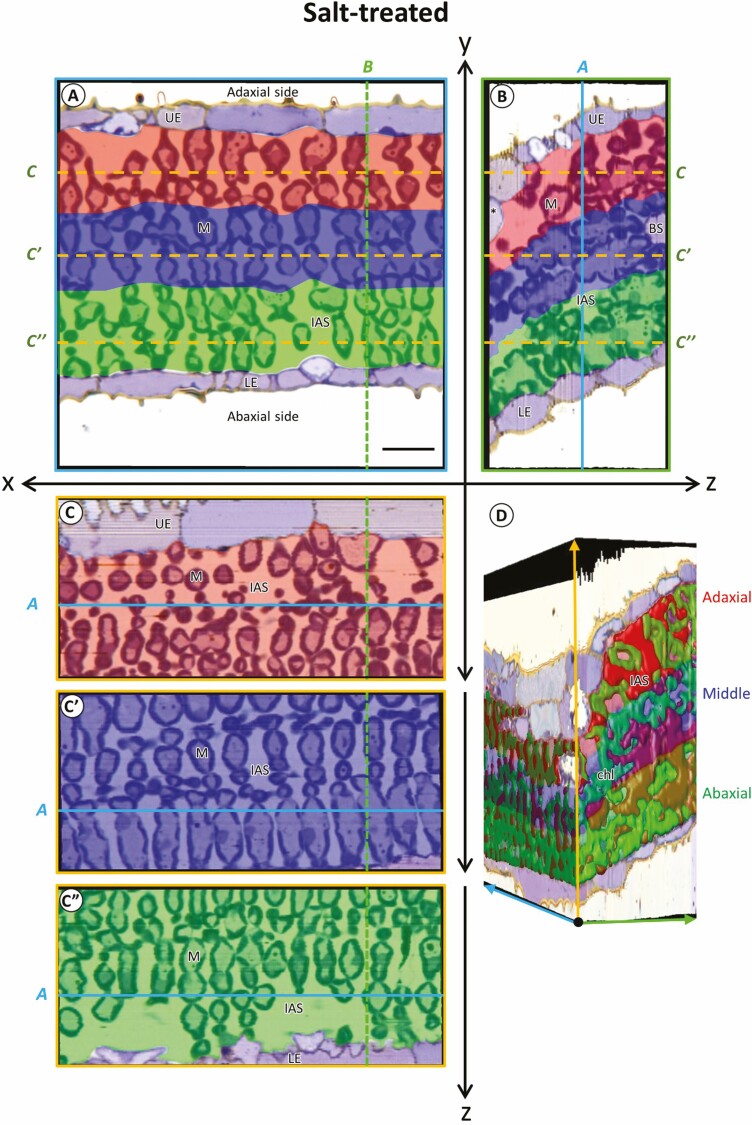
Orthogonal images of divided layers of mesophyll tissue of leaf blade in salt-treated rice. (A) Longitudinal image (*xy*). (B, C, Cʹ, C″) Orthogonal slice images of virtually imaged by volume rendering. (B) Transverse view (*yz*) of the image stacks, (C, Cʹ, C″) paradermal view (*xz*) of the image stacks. (D) Stacked view of the intercellular airspace and chloroplast in layers of leaf tissue. Each slice plane (A, B and C) crossed the lines marked as ‘*a, b, c*’ in other orientation images. Asterisk (*), motor cell, BS, bundle sheath cell; chl, chloroplasts; IAS, intercellular airspace; LE, lower epidermis; M, mesophyll cells; UE, upper epidermis. The colour ‘ Red, Blue, and Green’ indicated adaxial, middle and abaxial layers of MCs. Cutting interval (*z*-steps) = 0.5 µm. Total number of cuttings = 110. Scale bar = 20 µm.

Serial sections of the leaf segments located next to photosynthesis measurement on the leaf blade of control and salt-treated plants were stacked and divided into three layers with an equal *y* axis (Figs. 2 and 3). The leaf thickness was measured from the stacked images and showed no significant difference between the control and salt-treated plants (Table 1). The reconstructed image on the orthogonal views of the salt-treated leaves appeared to have narrow IAS with dense cells compared to the control leaves (Figs. 2 and 3). However, the volume ratio of IAS per tissue, the porosity of tissue and the MC number was not significantly different between the control and salt-treated plants in whole tissue (Table 1). Although salt treatment for 4 days had no significant effect on the overall tissue structures (the porosity and the MC number), the *S*_c_/*S*_mes_ significantly decreased in the salt-treated plants (Table 1).

**Table 1.** Effect of salinity stress on anatomical measurement of positional mesophyll tissue in rice leaves.

Treatment	Control				Salt-treated				ANOVA		
Position	(Whole)	Ada	Mid	Aba	(Whole)	Ada	Mid	Aba	*T*	*L*	*T* × *L*
Leaf thickness (µm)	98.7 ± 2.4				95.4 ± 10.9						
*V*_IAS_/*V*_Tissue_ (%)	29.8 ± 1.8	30.6 ± 2.2	27.9 ± 2.9	31.4 ± 0.3	27.7 ± 2.5	28.3 ± 3.2	27.5 ± 2.8	28.6 ± 1.5	n.s.	n.s.	n.s.
*V*_chl_/*V*_Tissue_ (%)	31.8 ± 1.8	35.2 ± 2.2	27.8 ± 0.3	33.5 ± 1.2	44.6 ± 2.3^**^	49.1 ± 1.8	41.4 ± 3.2	47.1 ± 5.0	**	**	n.s.
MC number (cell)	167.7 ± 7.0	51.7 ± 4.5	58.3 ± 5.5	55.5 ± 0.3	198.1 ± 36.5	69.9 ± 21.3	58.6 ± 3.0	74.6 ± 18.6	*	n.s.	n.s.
Porosity (µm^3^ µm^–3^)	0.31 ± 0.04	0.30 ± 0.02	0.28 ± 0.03	0.31 ± 0.00	0.28 ± 0.03	0.28 ± 0.03	0.27 ± 0.03	0.28 ± 0.02	n.s.	n.s.	n.s.
*S*_mes_ (µm^2^ µm^–2^)	19.4 ± 1.7	6.7 ± 0.5	6.6 ± 0.3	6.5 ± 0.8	20.4 ± 1.1	7.1 ± 0.7	7.3 ± 0.05	7.6 ± 0.3	**	n.s.	n.s.
*S*_c_ (µm^2^ µm^–2^)	18.3 ± 1.1	6.4 ± 0.4	6.0 ± 0.2	6.0 ± 0.7	17.9 ± 0.7	6.1 ± 0.8	5.8 ± 0.4	6.3 ± 0.5	n.s.	n.s.	n.s.
*S*_c_/*S*_mes_	0.95 ± 0.03	0.95 ± 0.02	0.91 ± 0.03	0.93 ± 0.02	0.88 ± 0.04^*^	0.87 ± 0.04	0.80 ± 0.06	0.84 ± 0.09	**	n.s.	n.s.

Notes: *V*_IAS_/*V*_Tissue_ is the volume ratio of intercellular airspace (IAS) to tissue excluded epidermis. *V*_chl_/*V*_Tissue_ is the volume ratio of chloroplasts (chl) to tissue excluded epidermis. The MC number is calculated from the volume of mesophyll tissue per mean size of the MC in each layer. Ada, adaxial; Mid, middle; Aba, abaxial layer in the leaf tissue as indicated in Figs. 2 and 3. The significant difference between whole leaf tissue of the control and salt-treated plants was compared by Student’s *t*-test (**P < 0.01, *P < 0.05). Effects of treatment (control and salt-treated) and layer (ada, mid, aba) were evaluated by ANOVA (**P < 0.01, *P < 0.05, n.s. *P* > 0.05). Values are mean ± s.d. (*n* = 3).

In the divided layers, the adaxial (Figs. 2 and 3; red-coloured) and the abaxial (Figs. 2 and 3; green-coloured) layers appeared to have wider IAS than in the middle layer (blue-coloured) in both control and salt-treated leaves (Figs. 2 and 3). However, the volume ratio of IAS per tissue, porosity, and *S*_c_ was not significantly different among the layers and the treatments (Table 1). In addition, the salinity stress affected MC number, *S*_mes_ and *S*_c_/*S*_me*s*_, but these structural parameters were not significantly different among the layers (Table 1). In contrast, the layers and treatments significantly affected the chloroplast volume ratio per tissue (Table 1).

### Effect of salinity stress on the shape and size of MC at different layers

The selected MC of each layer in the leaf tissues of plants in control and salt treatment were reconstructed into 3D representations and compared on the orthogonal views (Supporting Information—Figs. S2 and S3). On the different views, MCs exhibited significant profiles. The adaxial MC in the longitudinal views appeared to be long prolate with few projections in control and short prolate with few projections in salt-treated plants (Supporting Information—Figs. S2A and S3A). In the transverse view, the MC appeared to be wider prolate with well-developed lobes on the cell periphery in control and an oblate with shrinking lobes on the cell periphery in salt-treated plants (Supporting Information—Figs. S2B and S3B). In paradermal views, the MC appeared to be short prolate with few projections in control and long prolate with few projections in salt-treated plants (Supporting Information—Figs. S2C and S3C). The middle MC of the control and salt-treated appeared to be prolate with deep arms on the cell periphery in longitudinal and paradermal views (Supporting Information—Figs. S2D and F and S3D and F). In the transverse views, MC in both control and salt-treated leaves appeared to be oblate with several well-developed lobes on the cell periphery (Supporting Information—Figs. S2E and S3E). The abaxial MC in the longitudinal and paradermal views appeared to be long prolate with projections in control (Supporting Information—Fig. S2G and I) and appeared to be short prolate with projections in salt-treated plants (Supporting Information—Fig. S3G and I). In transverse views, the MC appeared to be oblate with smaller lobes on the cell periphery in control and less oblate in salt-treated plants (Supporting Information—Figs. S2H and S3H).

**Figure 4.**
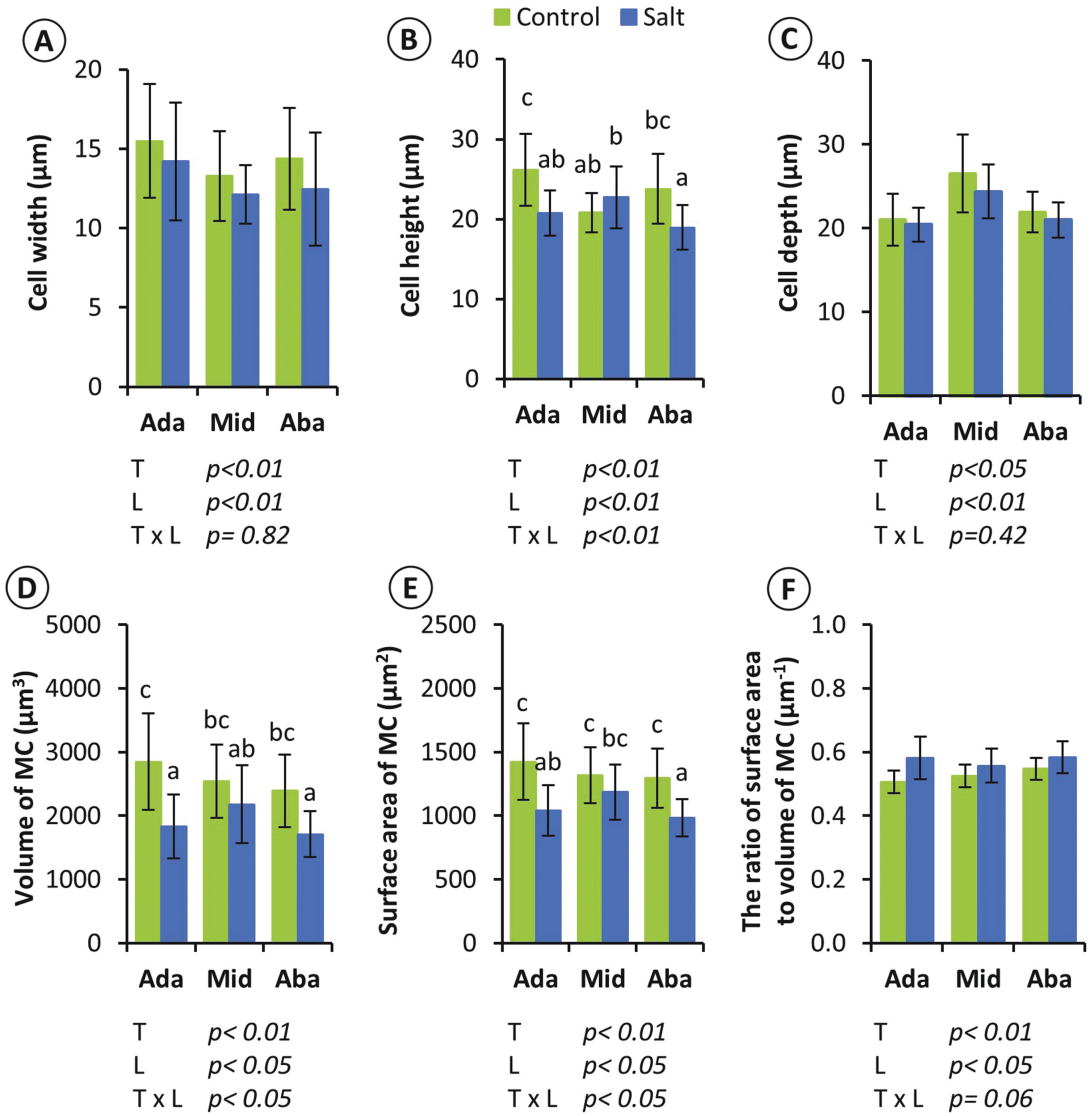
Quantitative comparison of MCs at different layers in control and salt-treated leaves. (a) Width, (b) height, (c) depth, (d) volume, (e) surface area and (f) the ratio of surface area to volume of MCs. Mean ± s.d. (*n* = 24, eight cells from three leaves). The results of a two‐way ANOVA are given in each panel (*T*, treatments; *L*, layers). For the parameters showing the significant interaction (*T* × *L*), each group (2 × 3 = 6) was compared by Tukey–Kramer multiple comparison tests, and different letters indicate significant differences (*P *< 0.05).

The shape and size of MC at each layer were calculated (Fig. 4, Supporting Information—Fig. S4). The cell width and depth were significantly different between treatments and layers (Fig. 4A and C). The cell height of the adaxial and abaxial MCs was significantly decreased in the salt-treated plants, while the middle layer was not significantly different (Fig. 4B). In addition, the feret diameters, which indicated the overall shape of the MCs, were significantly different between treatments and layers (Supporting Information—Fig. S4B‒D). Moreover, the volume and surface area of MC in the adaxial and abaxial layers significantly decreased after being treated with salinity stress (Fig. 4D and E). In contrast, the volume and surface area of the MC in the middle layer were not significantly different between the control and salt-treated plants (Fig. 4D and E). However, the surface area to volume ratio was significantly different between treatments and layers (Fig. 4F).

### Effect of salinity stress on the chloroplast structure in MC at different layers

**Figure 5.**
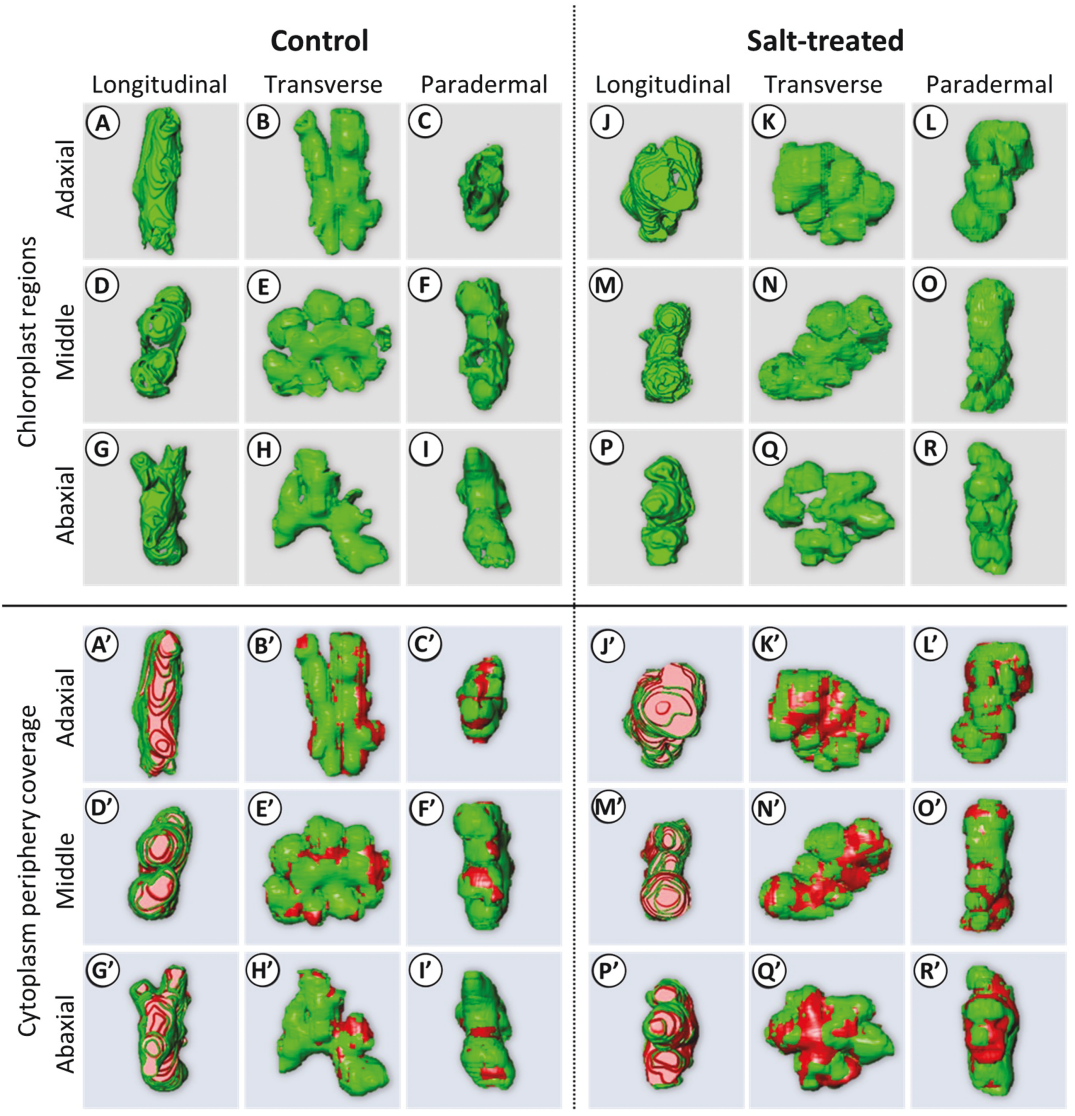
Reconstructed 3D representations of chloroplast regions and cytoplasm periphery coverage. (A–R) Chloroplast region in each MC. (Aʹ‒Rʹ) Cytoplasm periphery coverage by chloroplasts (green) and gap spaces not covered by chloroplasts (red) in each MC. Control plant (A–I, Aʹ–Iʹ), salt-treated plant (J–R, Jʹ–Rʹ). Adaxial layer (A–C, J–L), middle layer (D–F, M–O), (G–I, P–R) abaxial layer. (A, D, G; Aʹ, Dʹ, Gʹ) Longitudinal, (B, E, H; Bʹ, Eʹ, Hʹ) transverse (C, F, I; Cʹ, Fʹ, Iʹ) paradermal views. The represented chloroplasts of MCs in leaf tissue of control (Ada #3, Mid #2, Aba #2in Supporting Information—Fig. S2) and salt-treated (Ada #6, Mid #1, Aba#3 in Supporting Information—Fig. S3).

The 3D reconstruction of chloroplast regions in the selected MC of the control and salt-treated plants appeared to spread along the cell wall of MC in all layers (Fig. 5A‒R). The cytoplasm periphery coverage appeared to be higher in the control plants (Fig. 5A’‒I’) than in the salt-treated plants (Fig. 5J’‒R’) in all layers.

**Figure 6.**
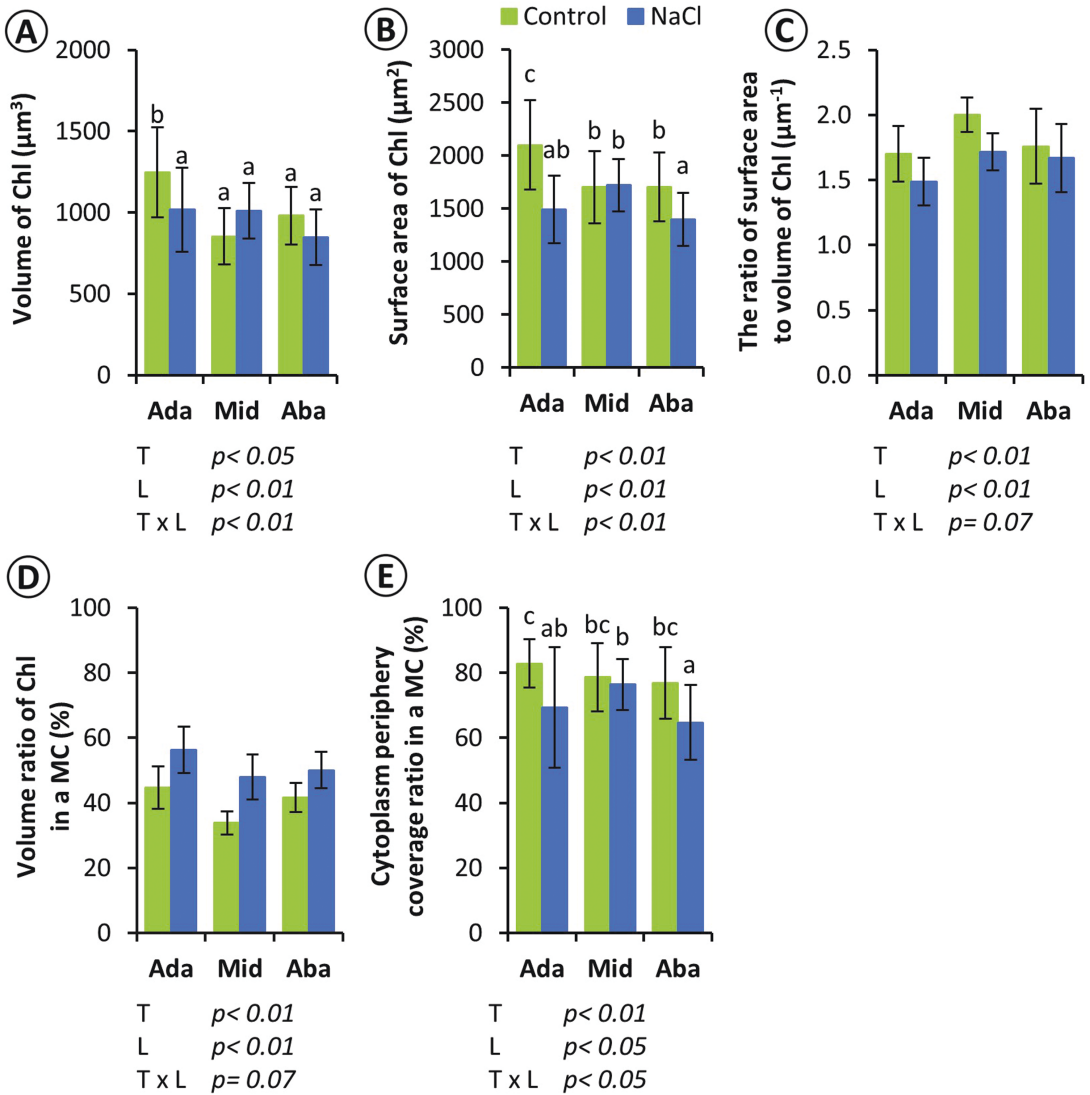
Quantitative comparison of positional mesophyll chloroplasts in control and salt-treated leaves. (a) Volume, (b) surface area, (c) ratio of surface area to volume of chloroplasts in MCs, (d) volume ratio of chloroplasts in an MC, (e) cytoplasm periphery coverage ratio in an MC. Chl, chloroplast; MC, mesophyll cell. Mean ± s.d. (*n* = 24, eight cells from three leaves). The results of a two‐way ANOVA are given in each panel (*T*, treatments; *L*, layers). For the parameters showing the significant interaction (*T* × *L*), each group (2 × 3 = 6) was compared by Tukey–Kramer multiple comparison tests, and different letters indicate significant differences (*P* < 0.05).

Under salinity stress, the volume of chloroplast regions of selected MC in the adaxial layer significantly decreased, while the middle and abaxial layers were not significantly different (Fig. 6A). The surface areas of chloroplasts regions in the adaxial and abaxial layers significantly decreased in the salt-treated plants and did not change in the middle layer (Fig. 6B). In addition, the surface area to the volume was significantly different between treatments and layers (Fig. 6C). Consistently, the volume ratio of chloroplasts in a selected MC was significantly different between the treatments and layers (Fig. 6D). Furthermore, the cytoplasm periphery coverage in a MC of salt-treated plants was lower than in the control plants at the adaxial and the abaxial layers, and there was no difference in the middle layer (Fig. 6E).

**Figure 7.**
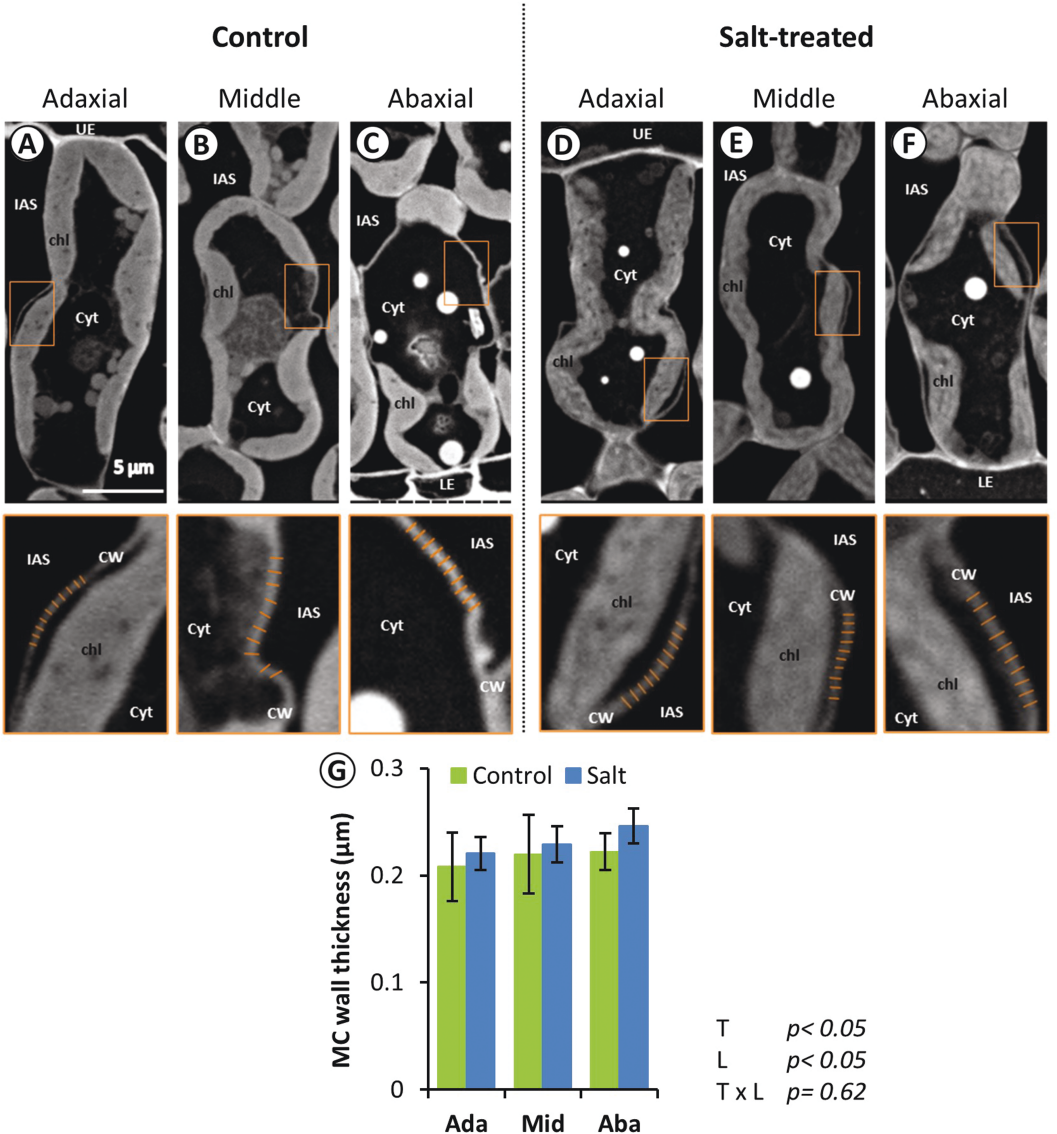
Cell wall thickness of MCs in each layer of the control and salt-treated leaves was observed by SEM. Adaxial (A and D), middle (B and E), abaxial layer (C and F). Control (A–C), Salt-treated plants (D–F). (g) MC wall thickness. The rectangle in each image indicated part measured the cell wall thickness. The image at the bottom of each panel indicated the magnified rectangle in the images. Chl, chloroplasts; Cyt, cytoplasm; CW, cell wall; IAS, intercellular airspace; LE, lower epidermis; UE, upper epidermis. Mean ± s.d. (*n* = 9, three cells from three leaves). The results of a two‐way ANOVA are given in each panel (*T*, treatments; *L*, layers).

The MC wall thickness was measured from each layer of the control and salt-treated plants (Fig. 7). In the control plants, almost all chloroplasts appeared to be in contact with the plasma membrane (Fig. 7A–C), while in the salt-treated plants, chloroplasts detaching from the plasma membrane were observed (Fig. 7D–F). Furthermore, the cell wall thickness was significantly different among the layers and higher in the salt-treated leaves than in the control leaves according to the two-way ANOVA (*P* < 0.05), whereas no significant difference was found between control and salt treatment in each layer (Fig. 7G).

## Discussion

### 3D structure of MC in rice leaf tissue

The 3D reconstruction following ssLM reveals that the MCs appeared to have different shapes, with significantly different cell widths, heights and depths depending on their layers (Supporting Information—Figs. S2, 4A–C and Supporting Information—S4C). The MC at the middle layer appeared to be an ellipsoid disc with several lobes on the cell periphery (Supporting Information—Fig. S2E), which is consistent with typical rice MC structure (Chonan, 1967; Sage & Sage, 2009; Oi *et al.*, 2017). However, compared to the typical MC structure, adaxial and abaxial MCs were not consistent in shape, which appeared to be a standing ellipsoid with well-developed lobes and an ellipsoid with smaller lobes, respectively (Supporting Information—Fig. S2A–C, Fig. S2G–I). Furthermore, the adaxial MCs showed significantly higher cell heights than the middle MCs (Fig. 4B), with the largest chloroplasts among the layers (Fig. 6A and B). These results support our hypothesis that rice MCs are not monotypic cells and indicate distinguished MC structures among the layers with greater diversity in the adaxial and abaxial layers. The greater lobes and chloroplasts in diverse MCs in these layers would increase the *S*_mes_ and *S*_c_.

### 3D structural changes of MC in response to salinity stress

In the salt-treated leaves, the photosynthetic parameters, *A*, *g*_*s*_ and *F*_*v*_/*F*_*m*_ were decreased (Fig. 1), indicating that salinity stress severely damaged the photosynthetic machinery. Since it has been reported that NaCl accumulation in leaves decreases photosynthetic activity (Yeo & Flowers 1986) and alters cell ultrastructure (Flexas *et al.* 2021), it is also possible that the decrease in photosynthetic capacity could be partly attributed to the decreased *S*_c_/*S*_mes_ (Table 1) and increased cell wall thickness (Fig. 7g). Changes in these parameters lead to lower CO_2_ diffusion conductance, which limits the CO_2_ diffuse from IAS into chloroplasts, as shown to occur in C_3_ plant leaves under salt stress conditions by Flexas *et al.* (2004). The results obtained in this study support our hypothesis by showing that the MC size decreased, especially at the adaxial and abaxial layers (Fig. 4D and E), which are closer to the leaf surface and receive more intense light than the middle layer (Supporting Information—Fig. S2).

Furthermore, the two phases of salinity stress lead to understanding the observed morphological changes. The first phase is that the rapid osmotic stress in saline conditions reduces the ability of plants to uptake water, resulting in slower leaf growth (Munns & Termaat 1986; Munns & Tester 2008). Although the leaves already reached the mature stage in this study, the osmotic stress would decrease the water uptake and the water potential of cells. The second phase is the ionic stress due to the high accumulation of Na^+^ in the mesophyll cell walls (Munns & Tester 2008), leading to the loss of turgor pressure and cell dehydration (Flowers & Yeo 1986). Since rice is susceptible to salinity, the Na^+^ accumulation in the shoot gradually increases within 3days (Yamane *et al.* 2009). The uneven distribution of water within the leaf is evident, with inner cells adjacent to vascular bundles tend to have more water supply compared to outer cells (Tanton & Crowdy 1972). The decrease in the size of MCs at the adaxial and abaxial layers under salinity stress might be caused by the combination of rapid osmotic stress and following ionic stress resulting from higher Na^+^ accumulation to these MCs. Thus, the reduction in the size of MCs located in outer layers is attributed to a greater decrease in turgor due to the increased salt content within these cells (Flowers & Yeo 1986). To reveal different Na^+^ concentrations between the adaxial and abaxial MCs, cryo-SEM X-ray microanalysis is a useful technique that can quantify the elemental concentration in various cell types of leaf tissues (Oi *et al.* 2022). Furthermore, under salinity stress, stomata close remarkably, therefore cuticular transpiration would be a major water flux from a leaf to atmosphere (Schuster *et al.* 2017). The significant decrease of the MC size at the adaxial and abaxial layers under salinity stress (Fig. 4D and E) might show more water loss from MCs adjacent to the epidermis compared to those in the middle layer. The different changes in MC size under salinity stress might be associated with the changes in the main pass of transpiration from through stomata to through epidermal cells.

Additionally, the treatment of 100 mM NaCl for 4 days in our study showed the significant effects of salinity stress to rice leaves, which means the higher salinity stress or longer period of salt-treatment would have more severe effects on the decreases of the MC size and photosynthetic parameters.

### Different responses of chloroplasts in leaf layers under salinity stress

The structural analysis of chloroplast regions in MC demonstrates that the chloroplasts in MCs of the middle layer distant from epidermal cells tend to be less affected by the salinity stress than those in adaxial and abaxial MCs closer to epidermal cells (Fig. 6), which was also consistent with the hypothesis that the combination of salinity stress and light cause more damage to the chloroplasts. The decrement of cytoplasm periphery coverage at adaxial and abaxial layers (Fig. 6E) and the detachment from the cell membrane (Fig. 7) suggest that deformation chloroplast surface negatively affected the photosynthetic traits through the decreased mesophyll CO_2_ conductance (Fig. 1).

Furthermore, the adaxial chloroplasts showed the greatest decrease in volume and surface area under the salinity stress (Fig. 6A and B), which may be attributed to the difference in light exposure between the leaf surfaces. The chloroplasts can change their intracellular positions by moving away from the light source to minimize photodamage under high-intensity light radiation (Yamada *et al.* 2009; Wada & Kong 2018), drought, salinity and osmotic stresses (Yamada *et al.* 2009). However, due to the large volume of the chloroplasts in intricated rice MC, they hardly changed their intracellular locations (Inoue & Shibata 1974; Kihara *et al.* 2020; Oi *et al.* 2020). The higher chloroplast volume in adaxial MC in the control plant (Fig. 6A) suggests that the limitation of the chloroplast movements in the cell might lead to being more strongly exposed to light than those at the middle and abaxial layers. It has been suggested that the structural change in the salt-stressed chloroplasts is dependent on light intensity (Mitsuya *et al.* 2003) and that the adaxial chloroplasts are more adapted to light than the abaxial ones (Terashima 1986). Our findings demonstrate the severe damage of the adaxial chloroplasts under stress conditions might be due to excess light exposure on the leaf surface, supported by decrement of the *F*_*v*_/*F*_*m*_, which was measured from the adaxial side. Further studies are required to reveal the gradient of the chloroplast response to light exposure along the adaxial–abaxial axis under salinity stress.

## Conclusion

The rice leaf tissue has different structures of the MC and chloroplasts with greater diversity at the adaxial and abaxial layers, and they respond differently to salinity stress. The adaxial and abaxial MCs were more severely affected than those in the middle layer, while the chloroplasts in the adaxial layer decreased the size and the cell periphery coverage area more than those in the middle and abaxial layers. Therefore, the MC at the adaxial layer seemed more sensitive to salinity stress, which may partly account for the decrease in photosynthetic capacity. To clarify the cause of the higher sensitivity of MC and chloroplast in the adaxial layer, and Na^+^ accumulation pattern and chloroplast properties along the adaxial–abaxial axis under salinity stress should be further investigated.

## Supporting Information

The following additional information is available in the online version of this article –

Figure S1. Rice plant growth under salinity stress for 4 days (25 days old). (A) Control plants. (B) Salt-treated plants.

Figure S2. Reconstructed 3D representations of MCs at different layers of leaf blade in the control rice. Adaxial (A–C), middle (D–F), abaxial layer (G–I). Longitudinal (A, D, G), transverse (B, E, H) and paradermal (C, F, I) views. The numbers indicate the order of MCs in leaf tissue.

Figure S3. Reconstructed 3D representations of MCs at different layers of leaf blade in the salt-treated rice. Adaxial (A–C), middle (D–F), abaxial layer (G–I). Longitudinal (A, D, G), transverse (B, E, H), paradermal views (C, F, I). The numbers indicate the order of MCs in leaf tissue.

Figure S4. Quantitative comparison of feret diameter of MCs at different layers in control and salt-treated leaves. (A) Illustration diagram showing maximum and minimum of feret diameters used to evaluate the shape of MC, (B) maximum feret diameter, (C) minimum feret diameter, (D) feret ratio (*F*_max_/*F*_min_). Mean ± s.d. (*n* = 24, eight cells from three leaves). The results of two‐way ANOVA are given in each panel (T; treatments, L; layers).

plae016_suppl_Supplementary_Data

## Data Availability

The data underlying this article are available in the online Supporting Information.
