# Supplementary material for: Structural changes of mesophyll cells in the rice leaf tissue in response to salinity stress based on the three-dimensional analysis
Source: AoB Plants. 2024 Apr 23;16(2):plae016. doi: 10.1093/aobpla/plae016 (PMC11059269; doi:10.1093/aobpla/plae016)
Supplement: plae016_suppl_Supplementary_Data [file plae016_suppl_supplementary_data.zip › SUPPORTING INFORMATION Captions.docx]

**SUPPORTING INFORMATION**

The following additional information is available in the online version of this article –

**Fig. S1 Rice plant growth under salinity stress for four days (25-days old).**

(A) Control plants. (B) Salt-treated plants.

**Fig. S2 Reconstructed 3D representations of MCs at different layers of leaf blade in the control rice.**

(A‒C) Adaxial, (D‒F) middle, (G‒I) abaxial layer. (A, D, G) Longitudinal, (B, E, H) transverse, (C, F, I) paradermal views. The numbers indicate the order of MCs in leaf tissue.

**Fig. S3 Reconstructed 3D representations of MCs at different layers of leaf blade in the salt-treated rice.**

(A‒C) Adaxial, (D‒F) middle, (G‒I) abaxial layer. (A, D, G) Longitudinal, (B, E, H) transverse, (C, F, I) paradermal views. The numbers indicate the order of MCs in leaf tissue.

**Fig. S4 Quantitative comparison of feret diameter of MCs at different layers in control and salt-treated leaves.**

(A) Illustration diagram showing maximum and minimum of feret diameters used to evaluate the shape of MC, (B) maximum feret diameter, (C) minimum feret diameter, (D) feret ratio (Fmax/Fmin). Mean ± s.d. (n = 24, 8 cells from 3 leaves). The results of two‐way ANOVA are given in each panel (T; treatments, L; layers).

Supporting data can be found in the following supplementary files:

Sup Info_Fig1. Data on growth and photosynthetic parameters

Sup Info_Fig4. Data on quantitative comparison of MCs at different layers

Sup Info_Fig6. Data on quantitative comparison of positional mesophyll chloroplasts

Sup Info_Fig7g. Data on cell wall thickness of MCs in each layer

Sup Info_FigS4. Data on quantitative comparison of feret diameter of MCs at different layers

Sup Info_Tab1. Data on anatomical properties of positional mesophyll tissue
