# Supplementary figures and images for: Structural changes of mesophyll cells in the rice leaf tissue in response to salinity stress based on the three-dimensional analysis
Source: AoB Plants. 2024 Apr 23;16(2):plae016. doi: 10.1093/aobpla/plae016 (PMC11059269; doi:10.1093/aobpla/plae016)

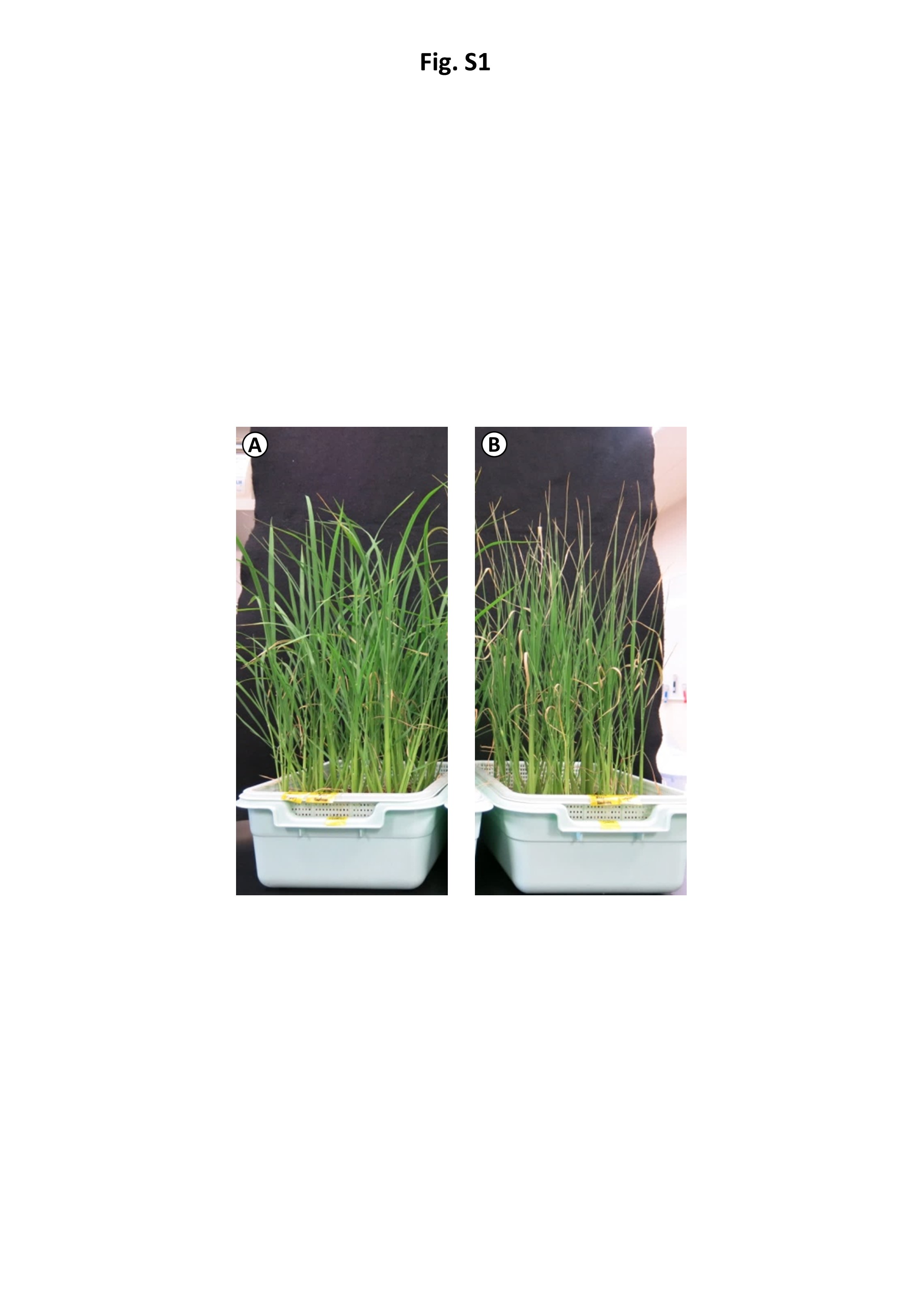

Supplement: plae016_suppl_Supplementary_Data [file plae016_suppl_supplementary_data.zip › Fig.S1.jpg]

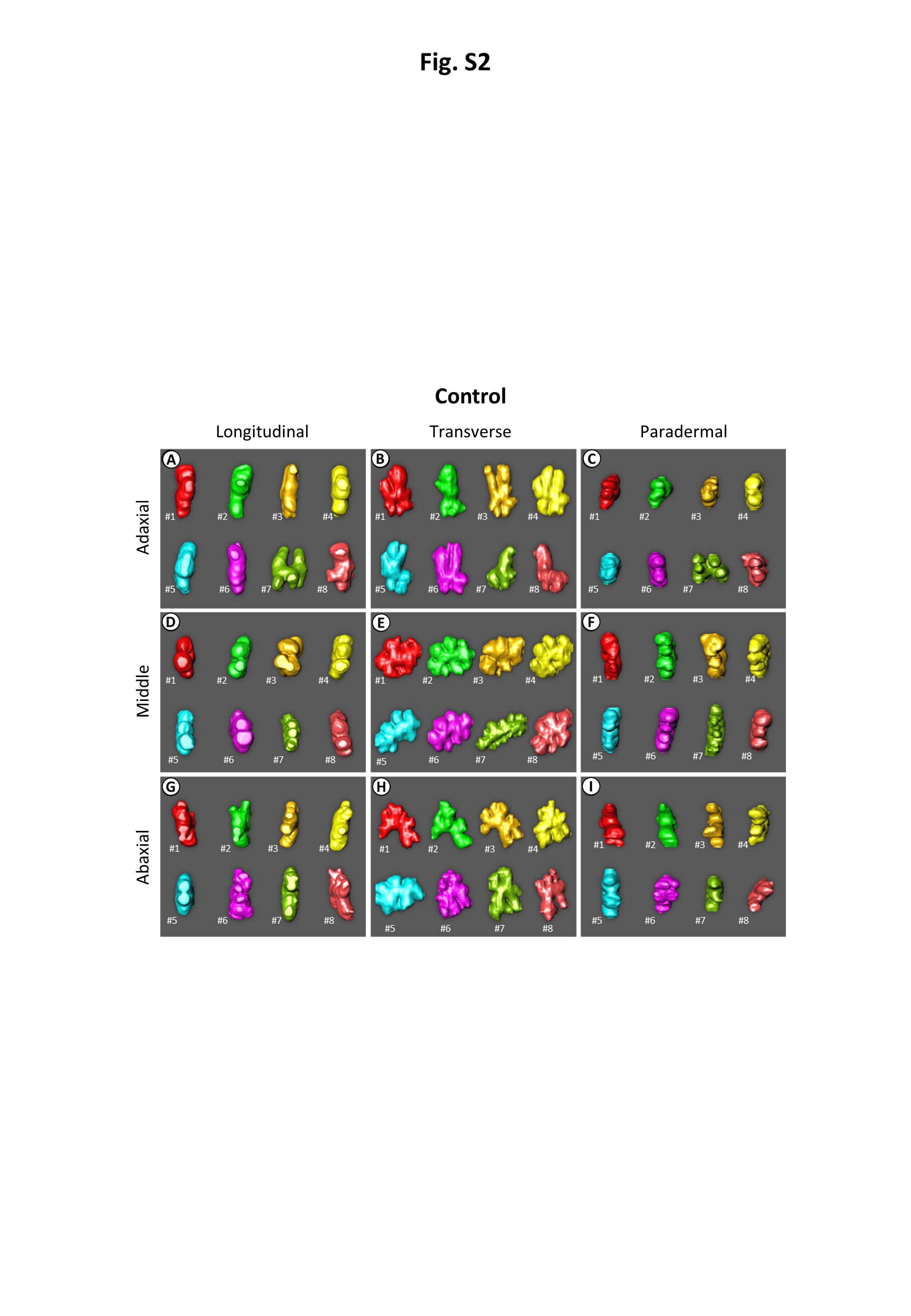

Supplement: plae016_suppl_Supplementary_Data [file plae016_suppl_supplementary_data.zip › Fig.S2.jpg]

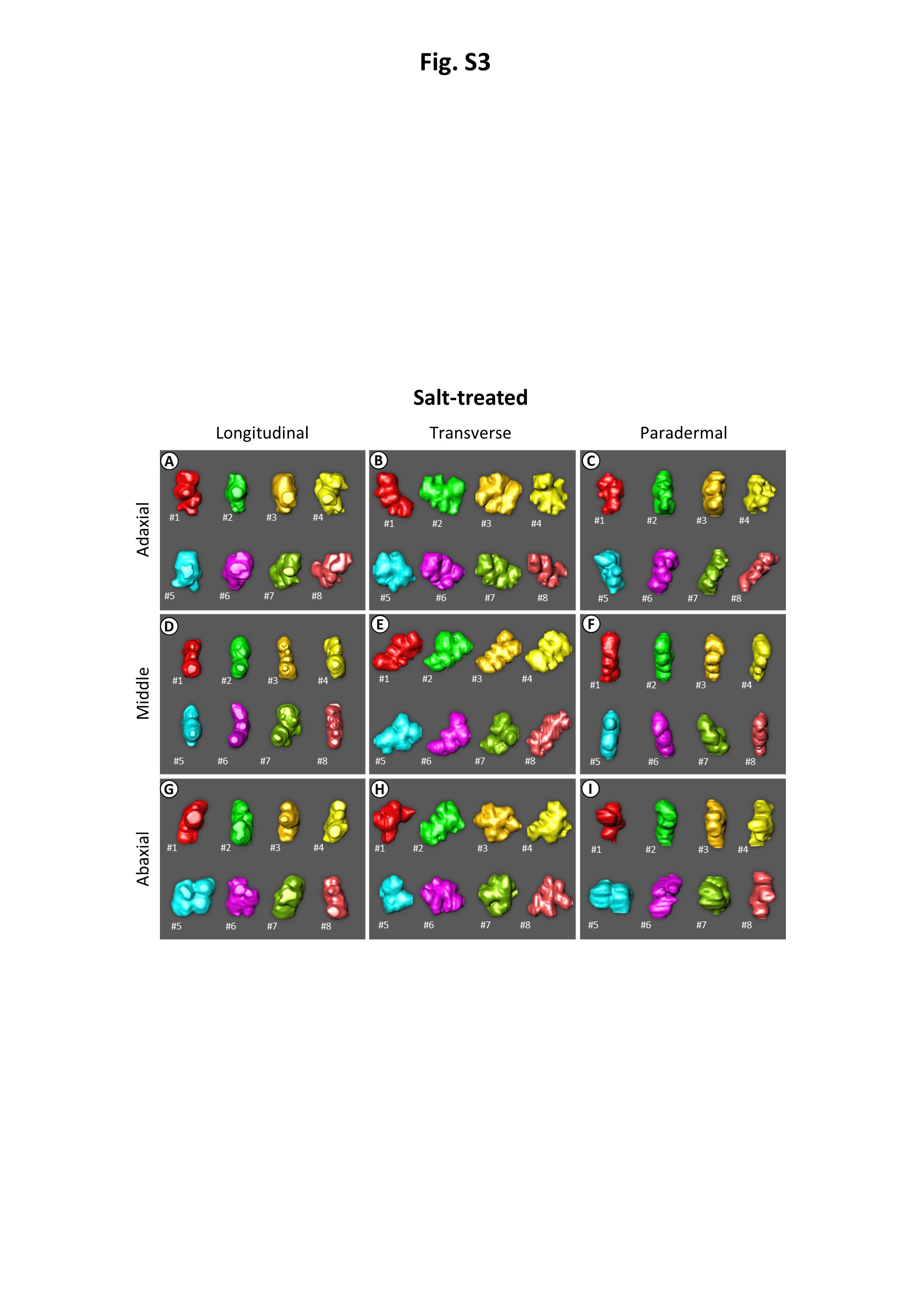

Supplement: plae016_suppl_Supplementary_Data [file plae016_suppl_supplementary_data.zip › Fig.S3.jpg]

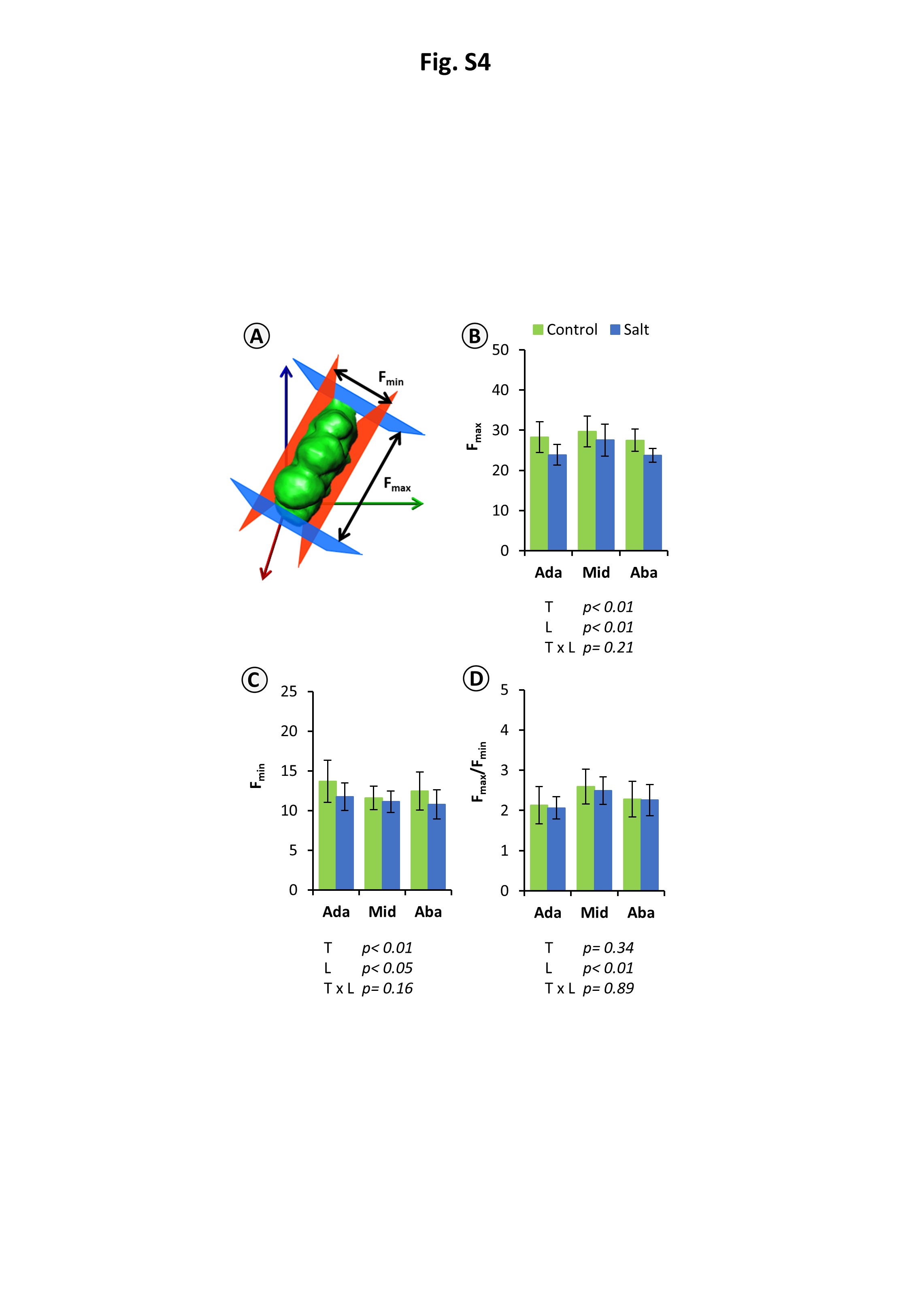

Supplement: plae016_suppl_Supplementary_Data [file plae016_suppl_supplementary_data.zip › Fig.S4.jpg]
